# Sequelae of Hospitalization for Diabetic Foot Ulcers at LASUTH Ikeja Lagos: A Prospective Observational Study

**DOI:** 10.3389/fcdhc.2022.889264

**Published:** 2022-08-18

**Authors:** Olufunmilayo Olubusola Adeleye, Adetutu Oluwatosin Williams, Akin Olusola Dada, Ejiofor T. Ugwu, Anthonia Okeoghene Ogbera, Olujimi Olanrewaju Sodipo

**Affiliations:** ^1^ Department of Medicine, Lagos State University Teaching Hospital, Ikeja, Nigeria; ^2^ Department of Medicine, Enugu State University of Science and Technology, Enugu, Nigeria

**Keywords:** diabetic foot ulcer, case fatality, clinical outcome, hospitalization, peripheral arterial disease

## Abstract

**Abstract:**

Diabetic foot ulcers (DFUs) remain important sequelae of diabetes (DM) which cause debilitating effects on the sufferer. The evolution of some aspects of epidemiology and the current clinical impact of DFUs was examined.

**Methods:**

A single-center prospective observational study. Study subjects were consecutively recruited.

**Results:**

Total medical admissions during the study period were 2288, 350 were DM related, out of these 112 were admitted for DFU. 32% of total DM admissions were for DFU. The mean age of the study subjects is 58 ± 11.0 range is from 35 years to 87 years. Males were slightly predominant (51.8%). Most of them were actively employed (92%), and the majority were in the 55 to 64 years age category. Most of them had not been diabetic for longer than 8 years (61%). The mean duration of DM is 8.32±7.27 years. The mean duration of ulcer at presentation was 72.0±138.13 days. The majority of the patients (80.3%) presented with severe (grades 3 to 5) ulcers, Wagner grade four was the most predominant. Regarding clinical outcome, 24 (24.7%) had an amputation, 3 of which were minor. The factor that was associated with amputation was concomitant heart failure – OR 6.00 CI 0.589-61.07, 0.498-4.856. Death occurred in 16 (18.4%). The factors associated with mortality were severe anemia OR 2.00 CI 0.65 – 6.113, severe renal impairment requiring dialysis OR 3.93 CI 0.232-66.5, concomitant stroke OR 8.42 CI 0.71-99.6, and peripheral arterial disease- OR 18.33 CI 2.27 -147 p-value- 0.006.

**Conclusion:**

The hallmark of DFU in this report is late presentation, it accounted for a significant proportion of the total medical admissions, although the case fatality of DFU reduced from previous reports from the center, mortality, and amputation rates are still unacceptably high. Concomittant heart failure was a factor of amputation. Mortality was associated with severe anemia, renal impairment and peripheral arterial disease.

## Introduction

Diabetic foot ulcers (DFUs) are common and potentially debilitating complications suffered by diabetic patients. The global prevalence of DFUs was recently estimated to be 6.3% (1). There is a significant burden imposed by DFUs on those affected and health systems globally (2). DFUs account for one of the most frequent causes of hospitalization in diabetic(DM) patients (3). Majority of these DFU admissions are due to neuropathy, peripheral vascular disease (PVD) and infections (4). Outcomes of DFUs are complex and not only influenced by comorbidities but also by the presence of diabetes-related chronic complications affecting other organs (5, 6). The most significant outcome of DFU apart from the death of the affected patient is amputation with its attendant long-term morbidity and predisposition to mortality. The outcome of foot ulcers also varies depending on the parameters considered as well as the health systems involved, for instance in Thailand, major amputations were performed in only 4.2% and 1.1% of mortality recorded for cases admitted for DFU (7). In Africa, DFUs result in infections, foot gangrene, prolonged hospitalization, and ultimately amputation or death (8). In a meta-analysis by Rigato et al (9), the overall prevalence of foot ulcers in Africa was 13% and was noted to have increased over time. In 2001, 15% of patients across Africa hospitalized for DFU had a major amputation and the overall mortality was 14% (9). Similarly in Nigeria, the outcome of hospitalization for DFU from a recent multicenter evaluation reported overall mortality of 21% and amputation of 35.4% (10, 11). It is important to report regional figures of DFU-related outcomes in a bid to not only audit clinical care provision, but also influence the equitable allocation of health financial and manpower resource. An earlier report from the current study center by Ogbera in 2007 observed that DFUs and hyperglycemic emergencies were common causes of death among hospitalized diabetic patients, the case fatality rate of DFUs was 28% (12). This study aims to identify the current status of outcomes of patients hospitalized for DFUs, document factors associated with amputation and mortality, and detect any changes in statistics related to DFUs compared with the earlier reports from the center.

## Methods

This is a cross-sectional, prospective observational study. The study was conducted at the Lagos State University Teaching Hospital (LASUTH). Consecutive patients hospitalized for DFUs at the medical wards, who gave verbal consent, were enrolled for 13 months. Those enrolled had type 2 DM. (only one type 1 Diabetic was admitted for DFU during the period).

Demographic, clinical, and laboratory data were recorded. Information sought included age, sex, diabetes duration, type of diabetes, visual impairment, smoking status, hospital or facility of usual care for diabetes before hospitalization(primary, secondary, tertiary, or alternative medicine) if currently employed.

Ulcer characteristics and risk factors for ulceration sought include; onset, whether traumatic or spontaneous, if walking barefoot/unshod, foot care education, history of amputation, ulcer site, Wagner grade, evidence of infection, presence of foot deformity, Neuropathy(Peripheral neuropathy was examined with Semmes Weinstein 10g monofilament or reduced vibration sense to 128hz tuning fork), and PVD.

Comorbid conditions sought include; hypertension, hyperglycemia, hypoglycemia, anemia, stroke, heart failure, shock, blood transfusion, and renal impairment with or without the requirement of renal replacement in form of hemodialysis.

Laboratory investigations documented include; glycated hemoglobin(HbA1c), white cell count, hematocrit, proteinuria, blood culture, Doppler ultrasound scan of lower limb vessels, and x-ray.

Management outcomes documented include ulcer healing and duration of same, amputation and extent of same, duration of hospitalization, whether discharged or demised during admission, and condition of ulcer after 3 months of follow-up.

Definition of study variables: Diabetic foot infections are defined according to the definition of the international working group on diabetes foot (13). Ulcers were graded with the Wagner grading of foot lesions (14). Ischemia was assessed clinically by palpation of the dorsalis pedis and posterior tibial arteries and Doppler ultrasound assessment of vascular flow. Inability to palpate the arteries or less than 50% narrowing was classified as PVD. Hypertension refers to blood pressure (BP) of 140/90mmHg and above or the use of blood pressure-lowering medications for the treatment of the same. Shock is systolic BP less than 90mmhg or diastolic BP less than 60mmhg. Anemia is hematocrit less than 40% in men and 36% in women. Hyperglycemic crisis in this study is a blood glucose level above 450mg/dl.

All study subjects were managed according to the hospital protocol in a multidisciplinary care setting.

Insulin was the only antidiabetic agent administered. The study subjects were followed up for 3 months after discharge or until death.

Data were analyzed using Statistical Package for Social Science version 23. Frequency and percentages were obtained for categorical variables. Comparison of proportions and association between categorical variables was done using chi-square and logistic regression. A p-value of less than 0.05 was considered significant.

Ethical clearance was obtained from the hospital’s research and ethics committee.

## Results

**Figure 1:**
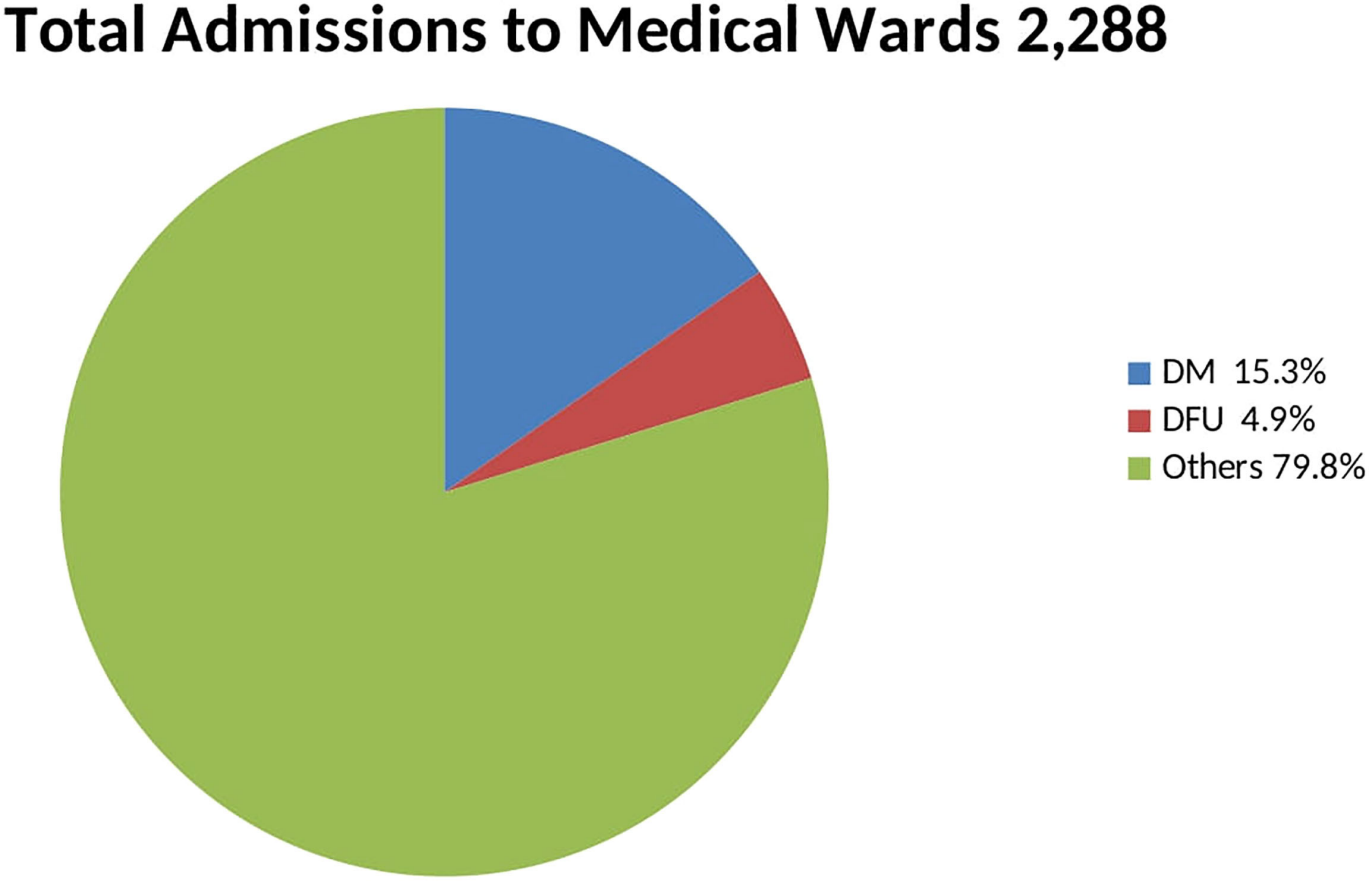
Pie chart showing proportion of admissions.

A total of 2,288 patients were admitted to the medical wards during the study period. Out of these, 350 were for DM-related causes. 112 patients with DFU were hospitalized and recruited (**Figure 1**). DFUs formed 32% of all DM-related admissions. Out of this number 11 patients left against medical advice during treatment. Some patients had missing data due to the inability to afford some of the investigations.

The mean age of the study subjects is 58 ± 11.0 ranging from 35 years to 87 years. Males were slightly predominant (51.8%). Most of them were actively employed (92%), and the majority were in the 55 to 64 years age category. Most of them had not been diabetic for longer than 8 years (61%). The mean duration of DM is 8.32 ± 7.27 years.

A majority (67%) had never received foot care education. Many of them had sought treatment from alternative practitioners and prayer houses before presenting to the hospital. The mean duration of ulcer at presentation was 72.0 ± 138.13 days. **Table 1** shows the demographic pattern, ulcer characteristics, and clinical parameters.

**Table 1:** Demographics, Ulcer characteristics and clinical parameters.

	Frequency(Percent)
***Age category***	
35 - 44	9 (8.0)
45 - 54	31 (27.7)
55 - 64	40 (35.7)
65 - 74	25 (22.3)
>=75	7 (6.3)
Males	58 (51.8)
***History of smoking***	
Current	1 (0.9)
Ex smoker	15 (13.4)
Never	96 (85.7)
Duration of DM<8yrs	68 (60.7)
No foot education	75 (67.0)
Spontaneous ulcer	61 (54.5)
No foot deformity	79 (70.5)
Visual impairment	25 (22.3)
Neuropathy	85 (78.0)
PAD	61 (54.5)
Walks Unshod	61 (54.5)
Hx of hypertension	53 (47.3)
Shock	2 (1.8)
Anaemia	58 (51.8)
hyperglycemia	26 (23.2)
hypoglycemia	23 (20.5)
Stroke	6 (5.4)
Heart failure	4 (3.6)
Renal impairment	26 (23.2)
Required dialysis	2 (1.8)
Required Blood transf	44 (39.3)
Ulcer healed during admission	11 (9.8)

The majority of the patients (80.3%) presented with severe (grades 3 to 5) ulcers, Wagner grade four was the most predominant.

Neuropathy is a prominent causative factor as 78% had clinical evidence of neuropathy. PAD was observed in 54% of the subjects.

Glycemic control before hospitalization was poor as evidenced by the markedly deranged mean HbA1c of 9.9%.

**Figure 2:**
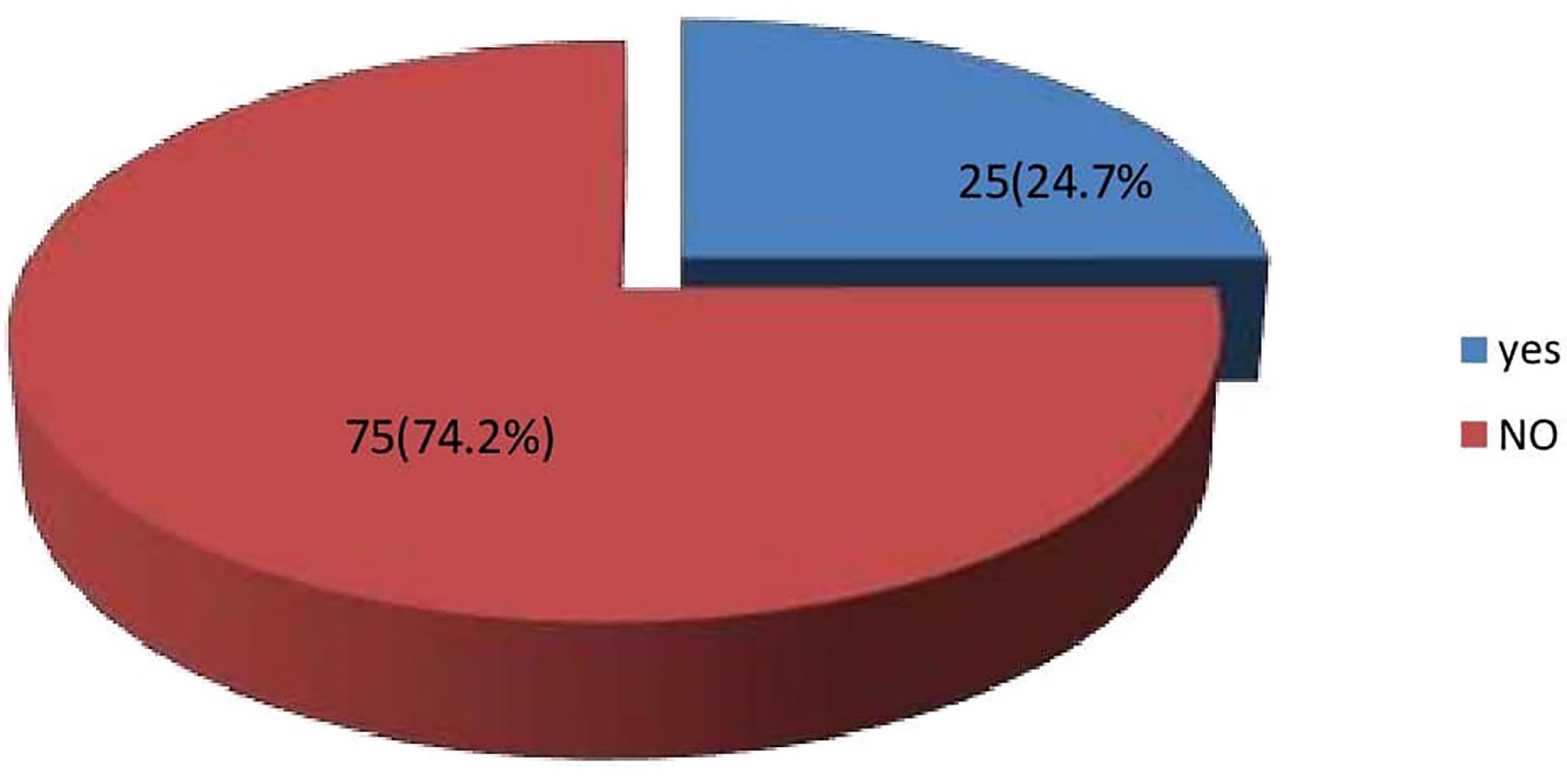
Frequency of Amputation.

**Figure 3:**
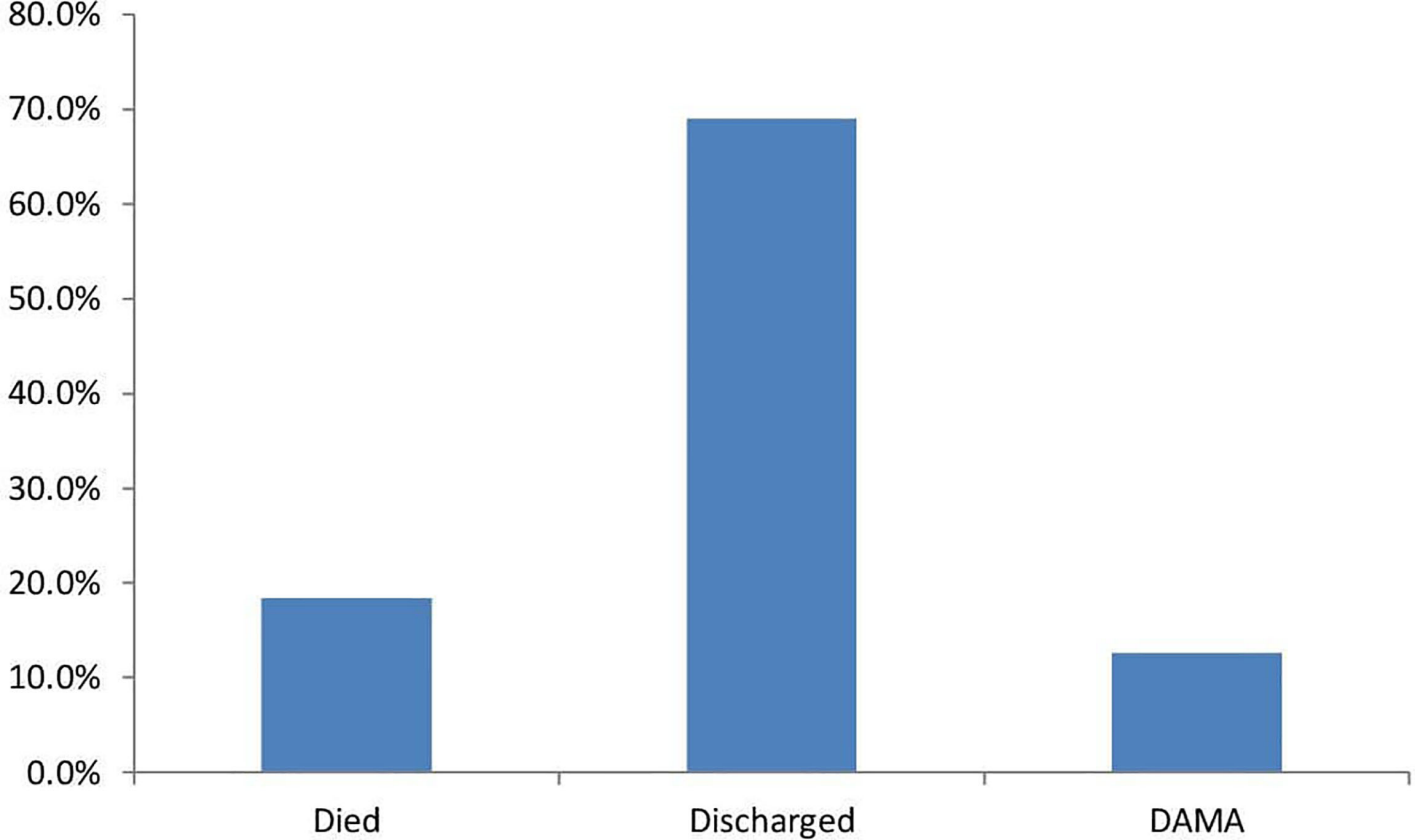
Outcome of hospitalization.

Regarding clinical outcome, 24 (24.7%) had an amputation, 3 of which were minor (**Figure 2**). The factors that were associated with amputation were, heart failure – OR 6.00 CI 0.589-61.07, PAD OR 1.200 CI 0.445-3.239., a requirement of blood transfusion OR 1.625 CI 0.607 -4.35, and renal impairment OR1.556 CI 0.498-4.856. Although these did not reach statistical significance (**Table 2**). Death occurred in 16 (18.4%) during hospitalization although the actual cause of death was not elucidated in this study **Figure 3**. The factors associated with mortality were the requirement of transfusion OR 2.00 CI 0.65 – 6.113, requiring dialysis OR 3.93 CI 0.232-66.5, concomitant stroke OR 8.42 CI 0.71-99.6, and PAD- OR 18.33 CI 2.27 -147 with a p-value of 0.006. Presentation to the hospital in hyperglycemic crisis was not a factor in mortality (**Table 3**).

**Table 2:** Relationship between clinical and demographic variables and Amputation.

	Amputation				
	Yesn (%)	Non (%)	P value	OR	95% C.I for OR
***History of Smoking***					
Never	21 (36.2)	37 (63.8)			
Current	0 (0.0)	1 (100.0)	NA	NA	NA
Ex-smoker	4 (36.4)	7 (63.6)	0.992	1.007	0.264 – 3.845
***Duration of DM***					
<8yrs	13 (34.2)	25 (65.8)	0.775	0.867	0.325 – 2.310
≥ 8yrs	12 (37.5)	20 (62.5)			
***Foot care education***					
Yes	7 (33.3)	14 (66.7)	0.786	0.861	0.293 – 2.529
No	18 (36.7)	31 (63.3)			
***Onset of ulcer***					
Spontaneous	15 (36.6)	26 (63.4)	0.857	1.096	0.405 – 2.965
Traumatic	10 (34.5)	19 (65.5)			
***Ulcer duration***					
>1 month	14 (35.9)	25 (64.1)	0.971	1.018	0.380 – 2.725
≤1 month	11 (35.5)	20 (64.5)			
***Foot deformity***					
Yes	7 (31.8)	15 (68.2)	0.645	0.778	0.267 – 2.269
No	18 (37.5)	30 (62.5)			
***Visual impairment***					
Yes	5 (27.8)	13 (72.2)	0.417	0.615	0.190 – 1.989
No	20 (38.5)	32 (61.5)			
***Neuropathy***					
Yes	20 (36.4)	35 (63.6)	0.828	1.143	0.342 – 3.817
No	5 (33.3)	10 (66.7)			
***PAD***					
Yes	15 (37.5)	25 (62.5)	0.719	1.200	0.445 – 3.239
No	10 (33.3)	20 (66.7)			
***Hypertension***					
Yes	11 (33.3)	22 (66.7)	0.695	0.821	0.308 – 2.194
No	14 (37.8)	23 (62.2)			
***Shock***					
Yes	2 (100.0)	0 (0.0)	NA	NA	NA
No	23 (33.8)	45 (66.2)			
***Anemia***					
Yes	15 (38.5)	24 (61.5)	0.591	1.312	0.487 – 3.538
No	10 (32.3)	21 (67.7)			
***Hyerglycemic crisis***					
Yes	4 (25.0)	12 (75.0)	0.313	0.524	0.149 – 1.841
No	21 (38.9)	33 (61.1)			
***Hypoglycemia***					
Yes	4 (25.0)	10 (71.4)	0.535	0.667	0.185 – 2.397
No	21 (37.5)	35 (62.5)			
***Stroke***					
Yes	1 (25.0)	3 (75.0)	0.649	0.583	0.057 – 5.924
No	24 (36.4)	42 (63.6)			
***Heart failure***					
Yes	3 (75.0)	1 (25.0)	0.130	6.000	0.589 – 61.073
No	22 (33.3)	44 (66.7)			
***Renal impairment***					
Yes	7 (43.8)	9 (56.3)	0.447	1.556	0.498 – 4.856
No	18 (33.3)	36 (66.7)			
***Required dialysis***					
Yes	1 (50.0)	1 (50.0)	0.673	1.833	0.110 – 30.637
No	24 (35.3)	44 (64.7)			
***Required blood transfusion***					
Yes	13 (41.9)	18 (58.1)	0.334	1.625	0.607 – 4.354
No	12 (30.8)	27 (69.2)			

**Table 3:** Relationship between some demographic and clinical variables and mortality.

	Outcome				
	Diedn (%)	Dischargedn (%)	P value	OR	95% C.I for OR
***History of Smoking***					
Never	14 (21.9)	50 (78.1)			
Current	0 (0.0)	1 (100.0)	NA	NA	NA
Ex-smoker	2 (18.2)	9 (81.8)	0.783	0.794	0.154 – 4.103
***Duration of DM***					
<8yrs	8 (17.4)	38 (82.6)	0.335	0.579	0.190 – 1.760
≥ 8yrs	8 (26.7)	22 (73.3)			
***Onset of ulcer***					
Spontaneous	8 (19.5)	33 (80.5)	0.722	0.818	0.271 – 2.468
Traumatic	8 (22.9)	27 (77.1)			
***Ulcer duration***					
>1 month	10 (25.6)	29 (74.4)	0.317	1.782	0.575 – 5.525
≤1 month	6 (16.2)	31 (83.8)			
***Neuropathy***					
Yes	12 (22.2)	42 (77.8)	0.696	1.286	0.365 – 4.529
No	4 (18.2)	18 (81.8)			
***PAD***					
Yes	15 (35.7)	27 (64.3)	0.006	18.333	2.274 – 147.803
No	1 (2.9)	33 (97.1)			
***Hypertension***					
Yes	10 (30.3)	23 (69.7)	0.089	2.681	0.859 – 8.367
No	6 (14.0)	37 (86.0)			
***Shock***					
Yes	0 (0.0)	2 (100.0)	NA	NA	NA
No	16 (21.6)	58 (78.4)			
***Anemia***					
Yes	12 (27.9)	31 (72.1)	0.103	2.806	0.812 – 9.695
No	4 (12.1)	29 (87.9)			
***Hyerglycemic crisis***					
Yes	0 (0.0)	20 (100.0)	NA	NA	NA
No	16 (28.6)	40 (71.4)			
***Hypoglycemia***					
Yes	6 (31.6)	13 (68.4)	0.200	2.169	0.664 – 7.087
No	10 (17.5)	47 (82.5)			
***Stroke***					
Yes	2 (66.7)	1 (33.3)	0.091	8.429	0.713 – 99.662
No	14 (19.2)	59 (80.8)			
***Heart failure***					
Yes	1 (25.0)	3 (75.0)	0.843	1.267	0.123 – 13.065
No	15 (20.8)	57 (79.2)			
***Renal impairment***					
Yes	7 (33.3)	14 (66.7)	0.111	2.556	0.805 – 8.110
No	9 (16.4)	46 (83.6)			
***Required dialysis***					
Yes	1 (50.0)	1 (50.0)	0.343	3.933	0.232 – 66.596
No	15 (20.3)	59 (79.7)			
***Required blood transfusion***					

## Discussion

Diabetic foot ulcers are a complication of DM with a far-reaching global impact in terms of cost, morbidity, and mortality. The increasing prevalence of type 2 DM in Africa and Nigeria implies that DM-related complications will also be on the upward trend. This study seeks to report the evolution of the outcome of hospitalization for DFU at one of the leading referral centers in the city of Lagos in the South West of Nigeria. Lagos is the most populous city in Nigeria and the second-most populous city in Africa (15). In the report by Ogbera in 2007 (12), at the same center, DFUs accounted for the second most common cause of death in the population studied(30%) with a case fatality of 25%. DFUs accounted for 32 percent of all DM-related admissions in this study. Mortality from DFUs was 18.4% and a case fatality rate of 14% indicated an improvement in outcome, although this value is still unacceptably high. The short duration of this study poses a challenge to ascertaining reasons for the modest improvement in outcome. However, the fact that the majority of the study population presented late to the hospital makes patient-related reasons a conjecture. A possible explanation may be better availability of diagnostic facilities and enhanced synergy among the multidisciplinary team involved with the management of these patients. In addition, the earlier study was conducted when LASUTH was newly elevated from a secondary to a tertiary health facility. Furthermore, the earlier study did not elaborate on the clinical and laboratory characteristics of the DFUs study population.

In the current report, there were more males hospitalized for DFU which agrees with previous studies (9, 16–19). Males may be involved with jobs and activities that are more physical placing them at higher risk of foot ulceration.

It is noteworthy that the majority of the study subjects had been diabetic for 8 years or less. Similarly, Ekpebegh and Eregie et al (20, 21) reported findings of a relatively shorter duration of DM in those hospitalized for DFU. This observation may be due to a higher number of patients walking barefoot or unshod which places them at higher risk of the earlier development of foot complications. Furthermore, being economically disadvantaged has been shown to cause poor medication adherence with consequent poor glycemic control that will predispose to the early development of complications. In contrast to this observation, in a report from Manchester, UK, the mean duration of DM in a cohort of 194 patients studied was 15.4 ± 9.9 years (22). Similarly, the report from Thailand reported a mean duration of DM of 12.2 years among patients hospitalized for DFU (7).

Another notable observation in this study is the late presentation to the hospital by the majority of the patients. The mean duration of the ulcer at presentation was two and a half months. Late presentation is defined as an ulcer presenting to the hospital after three weeks and is reported to be the hallmark of DFUs in some studies from Africa and other resource-poor countries (23, 24). Late presentation may be attributed to ignorance of the possible sequelae of prolonged foot ulceration, poor access to healthcare, seeking alternative remedies, and poverty. The majority of the subjects in this study pay out of pocket for healthcare services.

Although the etiology of DFUs is multifactorial, the major contributory factors are neuropathy and PVD. Other previously identified associated factors include foot deformity and poor glycemic control (25). In this study 78 percent of the study subjects had clinical evidence of neuropathy and 54.5 percent had evidence of PVD.

Twenty–five out of those who remained on admission had an amputation and 3 of these were minor giving an amputation rate of 24.7%. A recent study from the Republic of Benin, a close West African Country to Nigeria reported a prevalence of amputation of 33.2% among patients hospitalized for DFU (26). Interestingly; a study from Abuja Nigeria reported a drop in the prevalence of amputation to 10% after commencement of multidisciplinary care of patients with DFU at the center (27). The factors associated with amputation in this study were, PVD, heart failure, and neuropathy. The odds of amputation in patients who had concomitant heart failure are 6 times those who did not. Other studies have reported the association of PVD and neuropathy with amputation (2). Heart failure will further worsen the peripherally reduced perfusion from PVD and reduce the chances of healing of a foot ulcer. Other reported associations with amputation include increasing age, longer duration of DM, proteinuria, and elevated creatinine levels (28–31)

Mortality recorded in this study was 18.4%. The factors associated with mortality were severe anemia requiring blood transfusion, renal impairment requiring hemodialysis, hypertension, stroke, and PVD in ascending order of impact. The hospitalized DFU patient in this study who had a concomitant stroke had 8 times the odds of death and those with PVD had 18 times the odds of death. Boyko et al. reported the increased mortality and association with PVD evidenced by lower ABPI in the patient with DFU (32).

## Limitations

The major limitation of this study was its observational nature, thus data from patients who could not afford some investigations were missing. Moreover, those who left against medical advice could not be followed up implying that the mortality could have been higher than what is currently reported. Although all patients were managed as per protocol, some variability in individual clinical care received cannot be ruled out. The small sample size could also prevent the generalization of the outcome.

## Conclusions

The prevalence of foot ulcers is comparable to what has been reported across Africa. It is still marked by a late presentation to the hospital. The amputation rate is still unacceptably high. Case fatality of DFU however reduced from what was reported from this center in a previous study. The reason for this improvement would need to be further elucidated and confirmed with a longitudinal study. Patients admitted for DFU with concomitant comorbidities require prompt and vigorous attention to prevent mortality. Preventative measures such as advocacy at community levels, provision of foot care education, and setting up clinics dedicated to foot care may serve to reduce the prevalence and incidence of diabetic foot ulcers.

## Data Availability Statement

The raw data supporting the conclusions of this article will be made available by the authors, without undue reservation.

## Ethics Statement

The studies involving human participants were reviewed and approved by Lagos State university Teaching Hospital Ethical committee. Written informed consent for participation was not required for this study in accordance with the national legislation and the institutional requirements.

## Author Contributions

OA Conceptualized the study, did data interpretation and developed the manuscript. EU conceptualized and designed the protocol. AW, AD, and OS took part in data collection and reviewed the manuscript for intellectual content. AO reviewed the manuscript. All authors approved the final manuscript.

## Conflict of Interest

The authors declare that the research was conducted in the absence of any commercial or financial relationships that could be construed as a potential conflict of interest.

## Publisher’s Note

All claims expressed in this article are solely those of the authors and do not necessarily represent those of their affiliated organizations, or those of the publisher, the editors and the reviewers. Any product that may be evaluated in this article, or claim that may be made by its manufacturer, is not guaranteed or endorsed by the publisher.

## References

[1] Zhang P Lu J Jing Y Tang S Zhu D Bi Y. Global Epidemiology of Diabetic Foot Ulceration: A Systematic Review and Meta-Analysis. Ann Med (2017) 49(2):106–16. doi: 10.1080/07853890.2016.1231932 27585063

[2] Lo ZJ Surendra NK Saxena A Car J. Clinical and Economic Burden of Diabetic Foot Ulcers: A 5-Year Longitudinal Multi-Ethnic Cohort Study From the Tropics. Int Wound J (2021) 18(3):375–86. doi: 10.1111/iwj.13540 PMC8244009 33497545

[3] Nieto-Gil P Ortega-Avila AB Pardo-Rios M Cobo-Najar M Blasco-Garcia C Gijon-Nogueron G. Hospitalisation Cost of Patients With Diabetic Foot Ulcers in Valencia (Spain) in the Period 2009–2013: A Retrospective Descriptive Analysis. Int J Environ Res Public Health (2018) 15(9):1831. doi: 10.3390/ijerph15091831 30149552 PMC6163481

[4] Hicks CW Selvarajah S Mathioudakis N Sherman RL Hines KF Black JH III. Burden of Infected Diabetic Foot Ulcers on Hospital Admissions and Costs. Ann Vasc surg (2016) 33:149–58. doi: 10.1016/j.avsg.2015.11.025 PMC6048950 26907372

[5] Meloni M Izzo V Giurato L Uccioli L. A Complication of the Complications: The Complexity of Pathogenesis and the Role of Co-Morbidities in the Diabetic Foot Syndrome. In Diabetic foot syndrome (2018) 26:19–32. doi: 10.1159/000480041

[6] Gershater MA Löndahl M Nyberg P Larsson J Thörne J Eneroth M. Complexity of Factors Related to Outcome of Neuropathic and Neuroischaemic/Ischaemic Diabetic Foot Ulcers: A Cohort Study. Diabetologia (2009) 52(3):398–407. doi: 10.1007/s00125-008-1226-2 19037626

[7] Thewjitcharoen Y Krittiyawong S Porramatikul S Parksook W Chatapat L Watchareejirachot O. Outcomes of Hospitalized Diabetic Foot Patients in a Multi-Disciplinary Team Setting: Thailand’s Experience. J Clin Trans endocrinol (2014) 1(4):187–91. doi: 10.1016/j.jcte.2014.10.002 PMC5685051 29159100

[8] Abbas ZG Boulton AJ. Diabetic Foot Ulcer Disease in African Continent:’From Clinical Care to Implementation’–Review of Diabetic Foot in Last 60 Years–1960 to 2020. Diabetes Res Clin practice (2022) 183:109155. doi: 10.1016/j.diabres.2021.109155 34838640

[9] Rigato M Pizzol D Tiago A Putoto G Avogaro A Fadini GP. Characteristics, Prevalence, and Outcomes of Diabetic Foot Ulcers in Africa. A systemic Rev meta-analysis Diabetes Res Clin practice (2018) 142:63–73. doi: 10.1016/j.diabres.2018.05.016 29807105

[10] Adeleye OO Ugwu ET Gezawa ID Okpe I Ezeani I Enamino M. Predictors of Intra-Hospital Mortality in Patients With Diabetic Foot Ulcers in Nigeria: Data From the MEDFUN Study. BMC Endocr Disord (2020) 20(1):1–0. doi: 10.1186/s12902-020-00614-4 PMC7455894 32859203

[11] Ugwu E Adeleye O Gezawa I Okpe I Enamino M Ezeani I. Predictors of Lower Extremity Amputation in Patients With Diabetic Foot Ulcer: Findings From MEDFUN, a Multi-Center Observational Study. J foot ankle Res (2019) 12(1):1–8. doi: 10.1186/s13047-019-0345-y 31223342 PMC6570910

[12] Ogbera AO Chinenye S Onyekwere A Fasanmade O. Prognostic Indices of Diabetes Mortality. Ethnicity disease (2007) 17(4):721–5 18072385

[13] Lipsky BA Aragón-Sánchez J Diggle M Embil J Kono S Lavery L. IWGDF Guidance on the Diagnosis and Management of Foot Infections in Persons With Diabetes. Diabetes/metabolism Res Rev (2016) 32:45–74. doi: 10.1002/dmrr.2699 26386266

[14] Wagner FW Jr. The Dysvascular Foot: A System for Diagnosis and Treatment. Foot ankle (1981) 2(2):64–122. doi: 10.1177/107110078100200202 7319435

[15] Britannica, The Editors of Encyclopedia Britannica. Available at: https://www.britannica.com/place/lagos-Nigeria (Accessed 28 February 2022).

[16] Schofield H Haycocks S Robinson A Edmonds M Anderson SG Heald AH. Mortality in 98 Type 1 Diabetes Mellitus and Type 2 Diabetes Mellitus: Foot Ulcer Location is an Independent Risk Determinant. Diabetic Med (2021) 38(10):e14568. doi: 10.1111/dme.14568 33772856

[17] Ammar AS Khalid R Malik U Zeb M Abbas HM Khattak SB. Predictors of Lower Limb Amputations in Patients With Diabetic Foot Ulcers Presented in a Tertiary Care Hospital of Pakistan. J Pakistan Med. Assoc (2021) 71(9):2163–6. doi: 10.47391/JPMA.06-932 34580507

[18] Seghieri G Policardo L Gualdani E Francesconi P. Female Disadvantage in Risk of Adverse Outcomes After Incident Diabetic Foot Hospitalizations: A Population Cohort Study. Curr Diabetes Rev (2021) 18(6):e270821195904. doi: 10.2174/1573399817666210827121937 34455962

[19] Seghieri G De Bellis A Seghieri M Gualdani E Policardo L Franconi F. Gender Difference in the Risk of Adverse Outcomes After Diabetic Foot Disease: A Mini-Review. Curr Diabetes Rev (2021) 17(2):207–13. doi: 10.2174/1573399816666200716195600 32674734

[20] Ekpebegh CO Iwuala SO Fasanmade OA Ogbera AO Igumbor E Ohwovoriole AE. Diabetes Foot Ulceration in a Nigerian Hospital: in-Hospital Mortality in Relation to the Presenting Demographic, Clinical and Laboratory Features. Int Wound J (2009) 6(5):381–5. doi: 10.1111/j.1742-481X.2009.00627.x PMC7951567 19912395

[21] Eregie A Edo AE. Factors Associated With Diabetic Foot Ulcers in Benin–City, Nigeria. Nigerian Med (2008) 49(1):9–11.

[22] Oyibo SO Jude EB Tarawneh I Nguyen HC Armstrong DG Harkless LB. The Effects of Ulcer Size and Site, Patient’s Age, Sex and Type and Duration of Diabetes on the Outcome of Diabetic Foot Ulcers. Diabetic Med (2001) 18(2):133–8. doi: 10.1046/j.1464-5491.2001.00422.x 11251677

[23] Chalya PL Mabula JB Dass RM Kabangila R Jaka H Mchembe MD. Surgical Management of Diabetic Foot Ulcers: A Tanzanian University Teaching Hospital Experience. BMC Res notes (2011) 4(1):1–7. doi: 10.1186/1756-0500-4-365 21943342 PMC3189128

[24] Yammine K Akiki S Assi C Hayek MDF. Amputation as a First Treatment is Highly Associated With Late Presentation: An Underestimated Modifiable Major Risk Factor for Diabetic Foot Ulcer. Foot Ankle Specialist (2021) 30:19386400211067625. doi: 10.1177/19386400211067625 34967230

[25] Burgess JL Wyant WA Abdo Abujamra B Kirsner RS Jozic I. Diabetic Wound-Healing Science. Med (2021) 57(10):1072. doi: 10.3390/medicina57101072 PMC8539411 34684109

[26] Hodé AK Dédjan HA. Survival of Diabetic Patients Amputees for Diabetic Foot in the Endocrinology Department of the Hubert Koutoukou Maga National University Hospital Center (CNHU-HKM) in Cotonou, Benin. Med Metab Dis (2021) 16(1):80–86. doi: 10.1016/j.mmm.2021.09.003

[27] Anumah FO Mshelia-Reng R Abubakar A Sough T Asudo F Jamda MA. Management Outcome of Diabetic Foot Ulcers in a Teaching Hospital in Abuja, Nigeria. Age (Years) (2017) 54(13.04):21–90

[28] Akha O Kashi Z Makhlough A. Correlation Between Amputation of Diabetic Foot and Nephropathy. Iranian J Kidney diseases (2010) 4(1):27–31. 20081301

[29] Morbach S Furchert H Gröblinghoff U Hoffmeier H Kersten K Klauke GT. Long-Term Prognosis of Diabetic Foot Patients and Their Limbs: Amputation and Death Over the Course of a Decade. Diabetes Care (2012) 35(10):2021–7. doi: 10.2337/dc12-0200 PMC3447849 22815299

[30] Moulik PK Mtonga R Gill GV. Amputation and Mortality in New-Onset Diabetic Foot Ulcers Stratified by Etiology. Diabetes Care (2003) 26(2):491–4. doi: 10.2337/diacare.26.2.491 12547887

[31] Yesil S Akinci B Yener S Bayraktar F Karabay O Havitcioglu H. Predictors of Amputation in Diabetics With Foot Ulcer: Single Center Experience in a Large Turkish Cohort. Hormones (Athens Greece) (2009) 8(4):286–95. doi: 10.14310/horm.2002.1245 20045802

[32] Boyko EJ Ahroni JH Smith DG Davignon D. Increased Mortality Associated With Diabetic Foot Ulcer. Diabetic med: J Br Diabetic Assoc (1996) 13(11):967–72. doi: 10.1002/(SICI)1096-9136(199611)13:11<967::AID-DIA266>3.0.CO;2-K 8946155

